# Complementary Roles of the Classical and Lectin Complement Pathways in the Defense against *Aspergillus fumigatus*

**DOI:** 10.3389/fimmu.2016.00473

**Published:** 2016-11-03

**Authors:** Anne Rosbjerg, Ninette Genster, Katrine Pilely, Mikkel-Ole Skjoedt, Gregory L. Stahl, Peter Garred

**Affiliations:** ^1^Laboratory of Molecular Medicine, Department of Clinical Immunology, Faculty of Health and Medical Sciences, Rigshospitalet, University of Copenhagen, Copenhagen, Denmark; ^2^Department of Anesthesiology, Perioperative and Pain Medicine, Center for Experimental Therapeutics and Reperfusion Injury, Brigham and Women’s Hospital, Harvard Medical School, Boston, MA, USA

**Keywords:** complement, lectin pathway, MBL, IgM, *Aspergillus fumigatus*, immunocompromised

## Abstract

*Aspergillus fumigatus* infections are associated with a high mortality rate for immunocompromised patients. The complement system is considered to be important in protection against this fungus, yet the course of activation is unclear. The aim of this study was to unravel the role of the classical, lectin, and alternative pathways under both immunocompetent and immunocompromised conditions to provide a relevant dual-perspective on the response against *A. fumigatus*. Conidia (spores) from a clinical isolate of *A. fumigatus* were combined with various human serum types (including serum deficient of various complement components and serum from umbilical cord blood). We also combined this with inhibitors against C1q, mannose-binding lectin (MBL), and ficolin-2 before complement activation products and phagocytosis were detected by flow cytometry. Our results showed that alternative pathway amplified complement on *A. fumigatus*, but required classical and/or lectin pathway for initiation. In normal human serum, this initiation came primarily from the classical pathway. However, with a dysfunctional classical pathway (C1q-deficient serum), lectin pathway activated complement and mediated opsonophagocytosis through MBL. To model the antibody-decline in a compromised immune system, we used serum from normal umbilical cords and found MBL to be the key complement initiator. In another set of experiments, serum from patients with different kinds of immunoglobulin insufficiencies showed that the MBL lectin pathway contribution was highest in the samples with the lowest IgG/IgM binding. In conclusion, lectin pathway appears to be the primary route of complement activation in the absence of anti-*A. fumigatus* antibodies, whereas in a balanced immune state classical pathway is the main activator. This suggests a crucial role for the lectin pathway in innate immune protection against *A. fumigatus* in immunocompromised patients.

## Introduction

The fungus *Aspergillus fumigatus* has its natural habitat in soil where it decomposes organic debris and the fungus is usually non-pathogenic for immunocompetent humans. However, immunocompromised patients are highly susceptible to pulmonary invasion – a disease termed invasive pulmonary aspergillosis (IPA). IPA can turn into systemic dissemination when conidia (spores) mature into fungal hyphae breaching the pulmonary epithelia and reaching the blood stream. This exposes other organs like kidney, heart, and brain to fungal attack (1). With a mortality rate of 40–90%, IPA poses a serious threat to several patient groups suffering from immune demolishing diseases such as leukemia and AIDS or during immunosuppressive therapy used under organ transplantations (2).

Due to the small airborne conidia (2–3 μm), *A. fumigatus* is able to penetrate into the alveolar spaces and initiate an infection. The conidia are constantly present in our daily surroundings and exposure is practically inevitable (1). Azole-based drugs are commonly used as prophylaxis and treatment against *A. fumigatus* infections, but resistant strains of *A. fumigatus* are emerging, possibly due to agricultural use of azole-fungicides (3, 4). Thus, research covering new aspects of the immune response against *A. fumigatus* is important for future treatment alternatives.

As part of the innate immune defense, complement is an essential facilitator of opsonophagocytosis of invading pathogens. Complement is a system based on pattern-recognition molecules (PRMs) and protein cleavage cascades that rapidly intensify an anti-pathogenic response. Complement is initiated *via* three pathways: the lectin, the classical, and the alternative pathway. The lectin pathway works by direct binding of PRMs, named mannose-binding lectin (MBL), ficolins, and collectins, to pathogenic surfaces. PRM-associated serine proteases (MASPs) cleave C4 and C2, which lead to formation of the C3 convertase C4b2a that cleaves C3 into the strong opsonizing factor C3b. C1q, the classical pathway PRM, utilizes immunoglobulins as adaptors to bind pathogens and associated proteases (C1r/C1s) cleave C4 and C2 and mediate activation and deposition of C3b. Alternative pathway is activated by spontaneous hydrolysis of C3 and moreover works as a C3b-amplification loop. After C3 cleavage, all pathways unite into the terminal part of the cascade, which leads to formation of the lytic terminal complement complex (TCC) (5).

The organization of complement activation on *A. fumigatus* has not been fully elucidated and previous *in vitro* studies are based on the immunocompetent state. A compromised immune system is the leading cause of IPA, and thus we aimed to clarify the roles of the three complement pathways on *A. fumigatus* under both immunocompetent and immunocompromised conditions.

## Materials and Methods

### *A*. *fumigatus*

The *A. fumigatus* strain was obtained from a fatal case of IPA (a kind gift from Professor Romani from the Infectious Diseases Institute of the University of Perugia). *A. fumigatus* was grown on Sabouraud glucose agar with chloramphenicol (89579, Sigma-Aldrich) for 4 days at 37°C before resting conidia were harvested in PBS/0.025% Tween 20. Conidia were filtered to remove unwanted hyphae and afterward washed extensively before heat-inactivation for 15 min at 121°C in PBS. Aliquots of conidia were stored at −80°C. Concentrations applied: 5 × 10^7^ cells/ml for consumption assays and 1 × 10^7^ cells/ml for complement activation and phagocytosis assays.

### Primary Antibodies

For the experiments we used the following in-house produced antibodies (Abs): mouse anti-ficolin-2 mAb FCN219 (6) and mouse anti-ficolin-1 mAb cross-reacting with ficolin-2 (7). Moreover, we applied the following commercial Abs: mouse anti-MBL mAb (HYB 131-1, Bioporto Diagnotics, Gentofte, Denmark), rabbit anti-C1q pAb (A0136, Dako, Glostrup, Denmark), rabbit anti-IgM and anti-IgG pAbs (0425 and 0423, Dako), rabbit anti-C4c and -C3c pAbs (0369 and F0201, Dako), and mouse anti-TCC mAb clone aE11 (011-01, AntibodyChain, Utrecht, Netherlands). The isotype controls included were: mouse IgG1κ and IgG2α isotype controls (557273 and 555571, BD Biosciences, Albertslund, Denmark) and rabbit IgG isotype control (10500C, Invitrogen, Naerum, Denmark).

### Secondary Antibodies

The secondary Abs used for the experiments were: HRP-conjugated donkey anti-rabbit Ab (NA934V, GE Healthcare, Broendby, Denmark), HRP-conjugated rabbit anti-mouse pAb (P0260, Dako), HRP-conjugated streptavidin (RPN1231V, GE healthcare), FITC-conjugated goat anti-rabbit pAb (F1262, Sigma-Aldrich, Copenhagen, Denmark), and FITC-conjugated goat anti-mouse pAb (F0479, Dako).

### Inhibitors

Following specific Abs were used to inhibit the binding of ficolin-2, MBL, and C1q to their ligands: in-house produced anti-ficolin-2 inhibitory mAb FCN212 isotype IgG1 (unpublished), anti-MBL-inhibitory mAb 3F8 (8), and anti-C1q mAb clone CLB/C1q85 isotype IgG1 (MW1828, Sanquin, Amsterdam, Netherlands). We included mouse IgG1 isotype control (BD Biosciences) and anti-MBL mAb 1C10 (8) as mock-inhibitors.

### Proteins

Recombinant proteins were expressed and purified as previously described (9). In short, MBL and ficolin-2 were expressed in CHO-DG44 cells cultivated in RPMI 1640 medium (Sigma-Aldrich) supplemented with 10% FCS, 100 U/ml penicillin, 0.1 mg/ml streptomycin, 2 mM l-glutamine, and 200 nM methotrexate. Purification was performed with affinity chromatography using anti-ficolin mAb FCN219 for ficolin-2 purification or mannan–agarose for MBL purification. Purified C1q (A099) and purified C2 (A112) were purchased from CompTech, Tyler, TX, USA.

### Serum Samples

We applied three types of sera previously described from patients deficient in one of the following complement components: C2 (10), MBL (9), and C1q (11). Moreover, 17 venous blood samples and 23 umbilical cord blood samples were collected from healthy individuals. Blood was collected in no-additive glass vials, coagulated for 2 h/RT and centrifuged for 10 min at 2000 × *g*. Serum was stored at −80°C until experiments were performed. A normal human serum (NHS) pool was prepared from six individuals (three male/three female).

### Binding of Native MBL, Ficolin-2, and C1q Measured in Western Blotting

Conidia and NHS with inhibitors/mock-inhibitors were co-incubated for 1 h at 4°C end-over-end. After extensive washing, conidia were eluted with LDS sample buffer, and the total content was run on a 4–12% bis-Tris polyacrylamide gel under reducing conditions (Life Technologies). rficolin-2 (0.2 μg), rMBL (0.1 μg), and purified C1q (0.1 μg) were used as loading controls. Proteins were blotted onto polyvinylidene difluoride membranes (GE Healthcare) and the membranes were probed with anti-ficolin-1 mAb FCN106 (cross-react with ficolin-2)/rabbit anti-mouse-HRP, anti-MBL mAb HYB 131-1/rabbit anti-mouse-HRP, or anti-C1q pAb A0136/donkey anti-rabbit-HRP. Membranes were developed using SuperSignal West Femto Chemiluminescent Substrate (Thermo Scientific, Rockford, IL, USA).

### Complement Activation on *A. fumigatus* Measured in Flow Cytometry

Activation of complement on *A. fumigatus* was examined under various conditions (see below) and followed the same experimental procedure: 10^7^ conidia/ml were incubated in 10% human serum for 30 min at 37°C, then washed and stained with primary or isotype control Abs followed by FITC-conjugated secondary Abs in these combinations: anti-C4c pAb/goat anti-rabbit-FITC pAb; anti-C3c pAb/goat anti-rabbit-FITC pAb; anti-TCC mAb/goat anti-mouse-FITC pAb; rabbit IgG isotype/goat anti-rabbit-FITC pAb; and mouse IgG1 isotype/goat anti-mouse-FITC pAb. Ab staining was performed for 30 min at 4°C, and washing-steps were made in the specific assay-suitable dilution buffer. Deposition and formation of C4b, C3b, and TCC on the conidia was measured as mean fluorescence intensity (MFI) by flow cytometry (Gallios, Beckman Coulter) and data were analyzed using Kaluza software (Beckman Coulter).

### TBS/Ca^2+^ and TBS/Ca^2+^/Mg^2+^ Conditions

Complement activation on *A. fumigatus* was measured after incubation in the NHS pool diluted in either (I) TBS/Ca^2+^ [10 mM Tris–HCl, 150 mM NaCl, 2 mM CaCl_2_, 1% fetal calf serum heat-inactivated for 30 min at 56°C (HI-FCS) (pH 7.4)] or (II) TBS/Ca^2+^/Mg^2+^ [TBS, 2 mM CaCl_2_, 1 mM MgCl_2_, 1% HI-FCS (pH 7.4)].

### EDTA and Mg/EGTA Conditions

Complement activation on *A. fumigatus* was measured using the NHS pool diluted in the following buffers: (I) barbital buffer [5 mM barbital sodium, 145 mM NaCl, 2 mM CaCl_2_, 1 mM MgCl_2_, 1% HI-FCS (pH 7.4)], (II) EDTA buffer (TBS, 10 mM EDTA, 1% HI-FCS), or (III) Mg^2+^/EGTA buffer (TBS, 10 mM MgCl_2_, 10 mM EGTA, 1% HI-FCS).

### Reconstitution of C2-, MBL-, and C1q-Deficient Human Sera

*Aspergillus fumigatus* was added into the following sera: (I) C2-deficient serum diluted in barbital buffer and reconstituted with C2 (10 μg/ml), (II) MBL-deficient serum diluted in TBS/Ca^2+^ (to reduce alternative pathway interference) and reconstituted with MBL (10 μg/ml), and (III) C1q-deficient serum diluted in TBS/Ca^2+^ and reconstituted with C1q (10 μg/ml). Complement activation was measured using flow cytometry.

### Ficolin-2 and MBL Inhibition in C1q-Deficient Serum

Complement activation on *A. fumigatus* was measured in C1q-deficient serum, diluted in barbital buffer, using the following inhibitors (5 μg/ml): ficolin-2 inhibitor (FCN212), MBL inhibitor (3F8), or MBL mock-inhibitor (1C10).

### Opsonophagocytosis

Opsonization and phagocytosis was measured in an assay using FITC-conjugated conidia and isolated human neutrophils. FITC-conjugation was made by mixing FITC powder (F7250, Sigma-Aldrich) and conidia (5 × 10^−8^ μg/conidia) for 30 min end-over-end at room temperature followed by removal of unbound FITC by extensive washing. FITC-conjugated *A. fumigatus* conidia (1 × 10^7^/ml) were opsonized for 30 min at 37°C in 10% C1q-deficient serum including 10 μg/ml of either the MBL inhibitor (3F8) or mock-inhibitor (1C10). Opsonized conidia were washed and combined with human neutrophils isolated with Polymorphprep (Axis-Shield, Oslo, Norway) according to the manufacturer’s instructions. Neutrophils and conidia co-incubated for 30 min at 37°C in a cell ratio of 1:5. After washing the cells and before flow cytometric analysis, 50 μl tryphan blue was added to quench fluorescence from non-ingested conidia. FITC-positive neutrophils were identified by gating. Barbital buffer was used as dilution/washing buffer throughout the experiment.

We also performed fluorescence and differential interference contrast (DIC) imaging of neutrophils phagocytizing FITC-conjugated *A. fumigatus* conidia (using the same protocol) to get a visual impression of the process. We used Zeiss LSM 700 Axio Imager 2 with a Plan-Apochromat 63x/1.40 Oil DIC M27 objective and Carl Zeiss ZEN Blue edition software.

#### High- vs. Low-MBL UCS and NHS Pools

Complement activation on *A. fumigatus* was evaluated in normal and umbilical cord serum (UCS) samples divided into pools according to their relative MBL levels based on measurements from a sandwich ELISA assay (HYB 131-1/HYB 131-1*). Four serum pools were prepared: (I) “high-MBL” NHS (eight donors), (II) “high-MBL” UCS (eight donors), (III) “low-MBL” NHS (seven donors), and (IV) “low-MBL” UCS (seven donors). The MBL concentrations in the “low-MBL” pools were ~0.4 μg/ml and the “high-MBL” pools ~2 μg/ml. Each of the serum pools were diluted in barbital buffer and binding of IgG (1% serum) and IgM (5% serum) as well as deposition of C3b (10% serum) were measured in flow cytometry using these Ab combinations: anti-IgG pAb/goat anti-rabbit-FITC pAb; anti-IgM pAb/goat anti-rabbit-FITC pAb; anti-C3c pAb/goat anti-rabbit-FITC pAb; and rabbit IgG isotype/goat anti-rabbit-FITC pAb.

### C3b and MBL Correlation in UCS

The correlation between the two following parameters was evaluated: MBL concentrations in 23 umbilical cord EDTA plasma samples measured in ELISA (HYB 131-1/HYB 131-1*) and deposition of C3b on *A. fumigatus* measured in flow cytometry (as previously described).

### MBL and C1q Inhibition in NHS vs. UCS

An UCS pool (21 samples) and the NHS pool were mixed with the MBL inhibitor (5 μg/ml 3F8) or C1q-inhibitor (10 μg/ml CLB/C1q85) and mock-inhibitors (5 μg/ml 1C10 or 10 μg/ml mouse IgG1). The effect on complement activation was assessed by flow cytometry using barbital buffer as dilution buffer.

### Immunoglobulin Insufficiency

Serum samples were obtained from three patients with different immunological disorders: (I) IgA deficiency, (II) X-linked agammaglobulinemia (in IgG replacement therapy), and (III) common variable immunodeficiency (see Table 1). Samples were combined with MBL inhibitor (5 μg/ml 3F8) or C1q-inhibitor (10 μg/ml CLB/C1q85) and mock-inhibitors (5 μg/ml 1C10 or 10 μg/ml mouse IgG1), and the percent difference in C3b between inhibitor and mock-inhibitor treated serum was calculated from the flow cytometric MFI values.

**Table 1:** **C1q and MBL inhibition in sera from immunocompromised patients**.

Diagnosis	Treatment	IgG/IgM/IgA (g/L)	Bound IgG/IgM	C3b after MBL inhibition (%)	C3b after C1q inhibition (%)
IgA deficiency	No	16.2/0.6/<0.05	+++/+++	↑3	↓67
X-linked agammaglobulinemia	IgG therapy	10.3/<0.05/<0.05	++/−	↓61	↓18
Common variable immunodeficiency	No	2.6/<0.05/<0.05	+/+	↓75	↓7

*A. fumigatus (1 × 10^7^ conidia/ml) was incubated with sera from three patients with different immune disorders. Bound IgG and IgM were compared and rated relative to an NHS pool: >100%: +++; 50–100%: ++; <50%: +; 0%: −. The effect of C1q and MBL inhibition was evaluated based on the C3b deposition. The results show the mean% reduction in C3b deposition caused by the inhibitor [(1 − C3b with inhibitor/C3b with mock-inhibitor)%]. Serum levels of IgG, IgM, and IgA were obtained from the clinical records, and calculations of bound IgG/IgM and C3b were based on flow cytometric mean fluorescence intensity values. Inhibition experiments represent the means of three independent experiments. Downward-pointing arrows indicate a C3b reduction and upward-pointing arrows a C3b increase*.

### Statistics

Statistical analyses were performed with GraphPad Prism 6 (GraphPad Software, San Diego, CA, USA). The results represent the means ± SD of three independent experiments. For two-condition comparisons we used two-tailed Student’s *t*-test and for more than two conditions we used one-way ANOVA with Bonferroni’s multiple comparison correction. Correlation studies were evaluated using Spearman’s rank correlation. *p*-Values and multiplicity adjusted *p*-values: ns *p* > 0.05; *p ≤ 0.05; **p ≤ 0.01; ***p ≤ 0.001; ****p ≤ 0.0001.

### Ethical Approval

The study was approved by the regional Health Ethics Committee in the Capital Region of Denmark (reference no. H2-2011-133).

## Results

### Binding of Native MBL, Ficolin-2, and C1q to *A. fumigatus* with/without Specific Inhibitors

**Figure 1:**
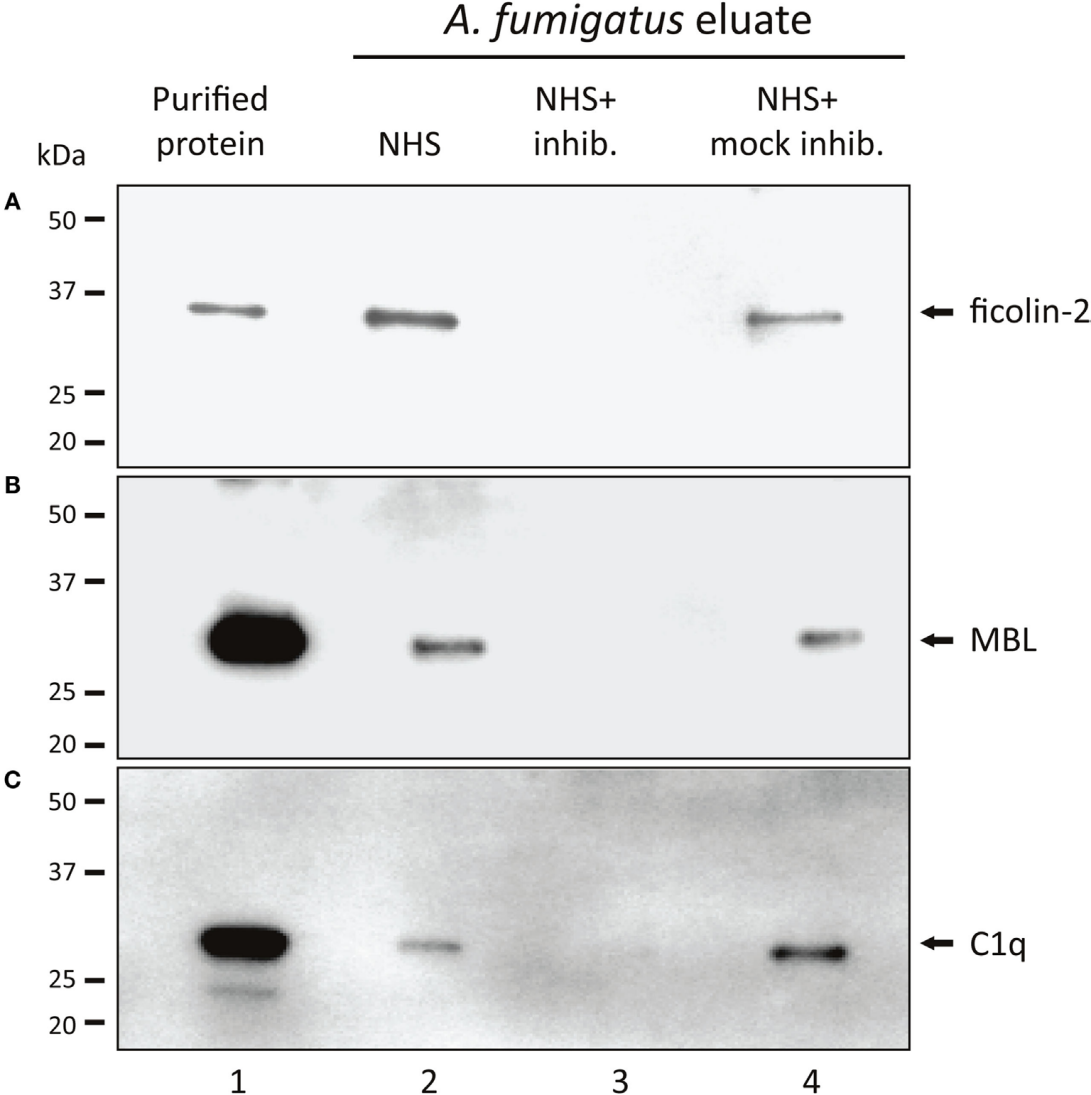
**Binding of native MBL, ficolin-2, and C1q to *A. fumigatus***. *A. fumigatus* conidia were incubated in NHS with ficolin-2, MBL, or C1q-inhibitor/mock-inhibitors, and eluates of the bound proteins were examined using SDS-PAGE and western blotting. **(A)** Ficolin-2, **(B)** MBL, and **(C)** C1q. Lane 1: purified rficolin-2, rMBL, and C1q. Lane 2: protein captured by *A. fumigatus* in NHS. Lane 3: captured protein in the presence of an inhibitor. Lane 4: captured protein in the presence of a mock-inhibitor.

Based on initial experiments, screening the complement PRMs C1q, MBL, ficolin-1, ficolin-2, and ficolin-3 for their ability to bind *A. fumigatus* (Figure S1 in Supplementary Material), the following PRMs were chosen as candidates for further studies: ficolin-2, MBL, and C1q. We confirmed the binding by incubating *A. fumigatus* with NHS followed by analyses of the *A. fumigatus* eluates with SDS-PAGE and Western blotting. Figures 1A–C shows the presence of ficolin-2, MBL, and C1q in the eluates (lane 2). We furthermore verified the efficacy of three specific inhibitory Abs targeting the PRM binding sites (lane 3) and proved the specificity using mock-inhibitory Abs as controls (lane 4).

### Alternative Pathway Amplification – Not Initiation

**Figure 2:**
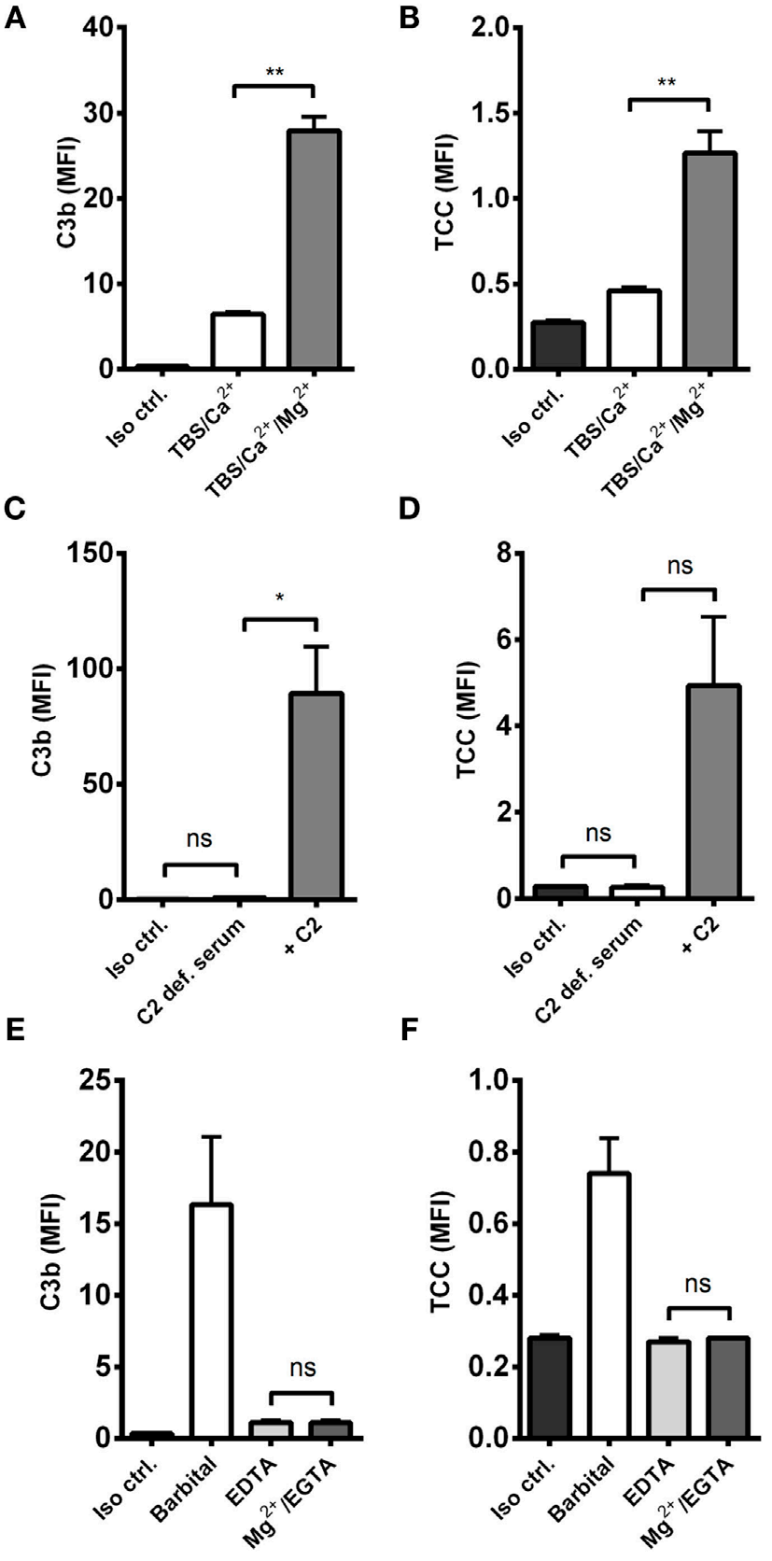
**Alternative pathway-mediated complement amplification on *A. fumigatus***. Complement activation was measured on *A. fumigatus* (1 × 10^7^ conidia/ml) after incubation in various buffers and sera. **(A,B)** NHS-generated C3b and TCC with/without Mg^2+^ in the dilution buffer. **(C,D)** C3b and TCC from C2-deficient serum with/without reconstitution of C2 **(E,F)** NHS-generated C3b and TCC under Mg^2+^/EGTA and EDTA conditions. Complement products were measured by flow cytometry and expressed as mean fluorescence intensity (MFI). Results represent the means of three independent experiments ± SD, *p ≤ 0.05, **p ≤ 0.01 (two-tailed paired Student’s *t*-test or one-way ANOVA, Bonferroni’s multiple comparison test).

The effect of alternative pathway on *A. fumigatus* was examined by combining *A. fumigatus* and NHS under two conditions: calcium-sufficient or both calcium- and magnesium-sufficient. The results clearly showed that magnesium amplified C3b and TCC, suggesting an alternative pathway-driven response (Figures 2A,B).

Next, we excluded the influence of classical and lectin pathway by measuring complement activation in human C2-deficient serum, naturally lacking the capacity to form classical/lectin pathway C3 convertase (C4b2a), and found that complement could not be activated without reconstituting C2 (Figures 2C,D).

**Figure 3:**
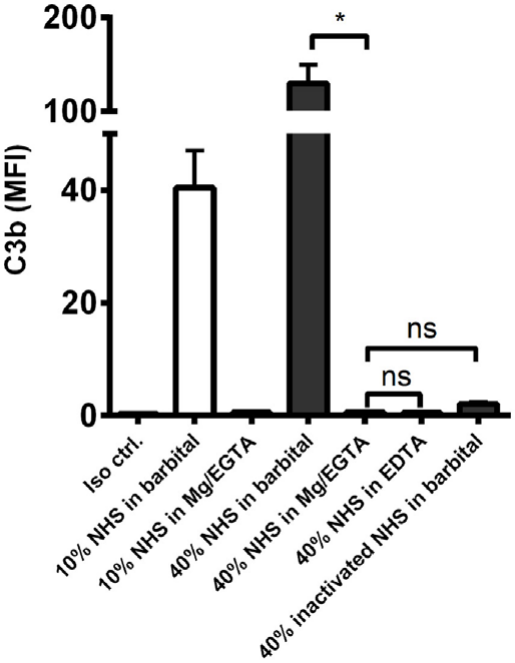
**Alternative pathway-mediated complement activation on *A. fumigatus* in 40% normal human serum**. C3b deposition was measured on *A. fumigatus* (1 × 10^7^ conidia/ml) after incubation in 10 and 40% NHS under following conditions: barbital buffer, 10 mM Mg/EGTA, and 10 mM EDTA. C3b deposition was measured by flow cytometry and expressed as mean fluorescence intensity (MFI). Results represent the means of three independent experiments ± SD, *p ≤ 0.05 (one-way ANOVA, Bonferroni’s multiple comparison test).

We then examined the complement response in NHS under magnesium-sufficient/calcium-deficient conditions (Mg^2+^/EGTA). We found that NHS diluted in Mg^2+^/EGTA facilitated the same levels of C3b and TCC as in EDTA, i.e., no downstream activation occurred when the classical and lectin pathways were excluded (Figures 2E,F). Increasing the serum concentration to 40% did not enable activation of the alternative pathway in Mg^2+^/EGTA either (Figure 3). Thus, taken together, Figures 2C–F and 3 shows that classical and/or lectin pathways are a prerequisite for complement initiation on *A. fumigatus*.

### Complement Activation in MBL- and C1q-Deficient Serum

**Figure 4:**
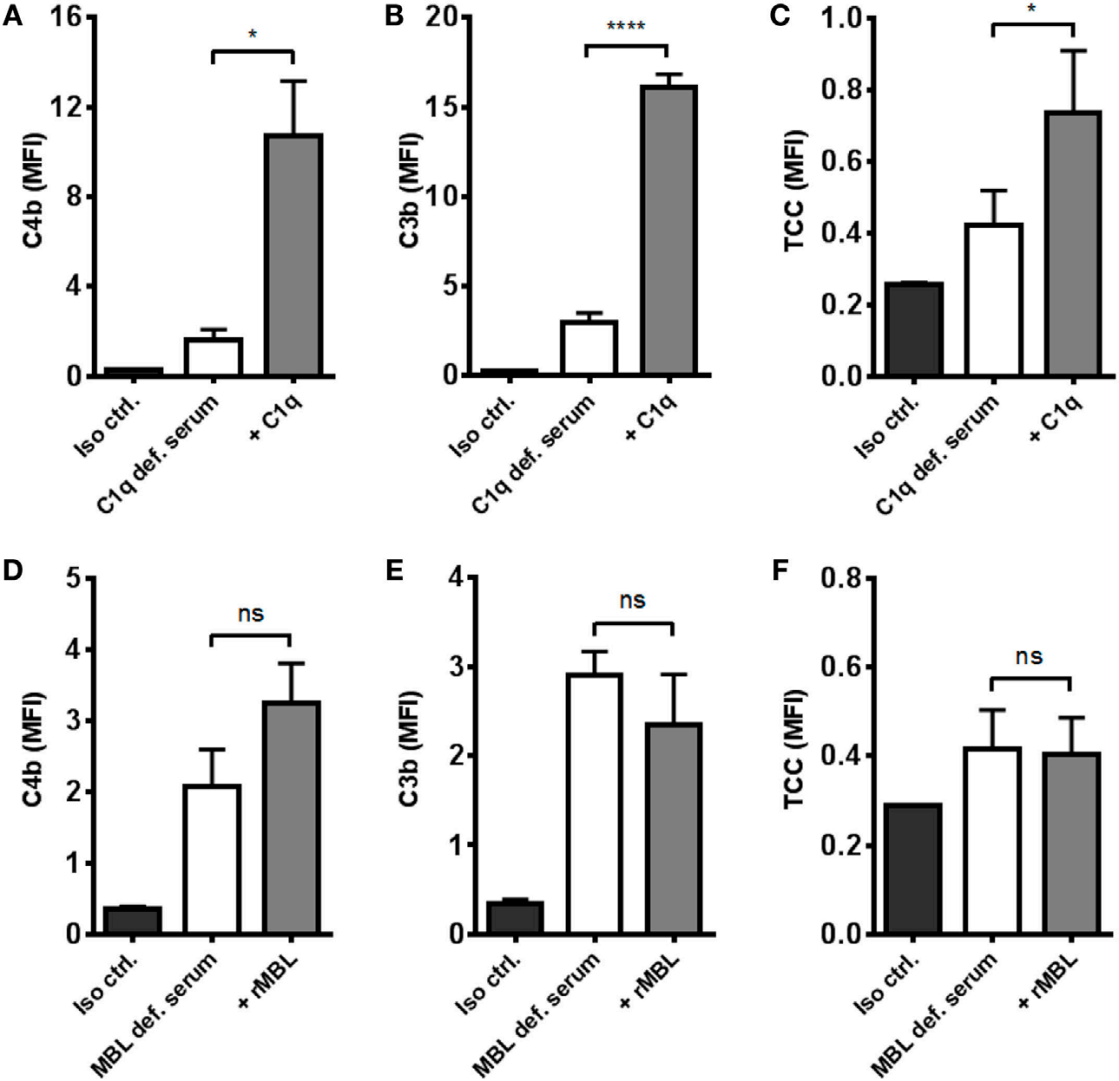
**Reconstitution of C1q- and MBL-deficient sera**. *A. fumigatus* (1 × 10^7^ conidia/ml) was added into **(A–C)** C1q- or **(D–F)** MBL-deficient serum diluted in TBS/Ca^2+^ (no Mg^2+^). Complement products were measured by flow cytometry and expressed as mean fluorescence intensity (MFI): **(A,D)** C4b, **(B,E)** C3b, and **(C,F)** TCC. Results represent the means of three independent experiments ± SD, *p ≤ 0.05, ****p ≤ 0.0001 (two-tailed paired Student’s *t*-test).

To distinguish between the contribution from the classical and the lectin pathways to complement activation, we tested the effect of reconstituting C1q- and MBL-deficient serum. By omitting magnesium in the dilution buffer, we excluded alternative pathway interference and focused on the two other pathways. Reconstitution of C1q-deficient serum significantly increased C4b, C3b, and TCC (Figures 4A–C). On the contrary, reconstitution of MBL-deficient serum did not affect activation of complement except for a non-significant increase in C4b deposition (Figures 4D–F). These results imply that the classical pathway is the dominant complement initiator in response to *A. fumigatus*.

### MBL and Ficolin-2 Inhibition in C1q-Deficient Serum

**Figure 5:**
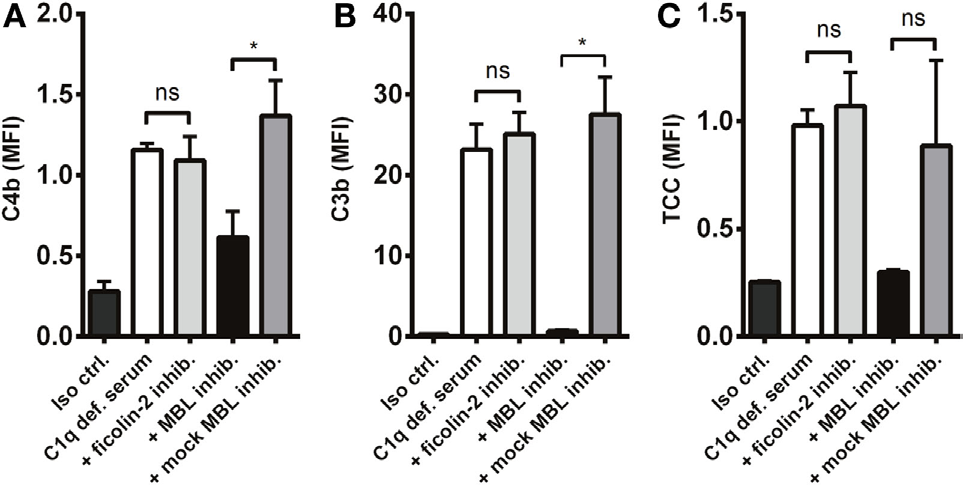
**MBL-mediated complement activation in the absence of the classical pathway**. *A. fumigatus* (1 × 10^7^ conidia/ml) was added into C1q-deficient serum plus ficolin-2 inhibitor, MBL inhibitor, or MBL mock-inhibitor. Complement products were measured by flow cytometry and expressed as mean fluorescence intensity (MFI): **(A)** C4b, **(B)** C3b, and **(C)** TCC. Results represent the means of three independent experiments ± SD, *p ≤ 0.05 (one-way ANOVA, Bonferroni’s multiple comparison test).

Next, we investigated the process of complement activation in the absence of the classical pathway. For this purpose, we applied C1q-deficient serum in combination with two lectin pathway inhibitors targeting MBL and ficolin-2. We found that complement was still activated in C1q-deficient serum and interestingly, inhibition of MBL and not ficolin-2 reduced C4b and C3b deposition on *A. fumigatus* (Figures 5A,B). We also observed a drop in TCC, although not statistically significant (Figure 5C). Thus, MBL drives the activation of complement under conditions with a dysfunctional classical pathway.

### MBL-Mediated Opsonophagocytosis

**Figure 6:**
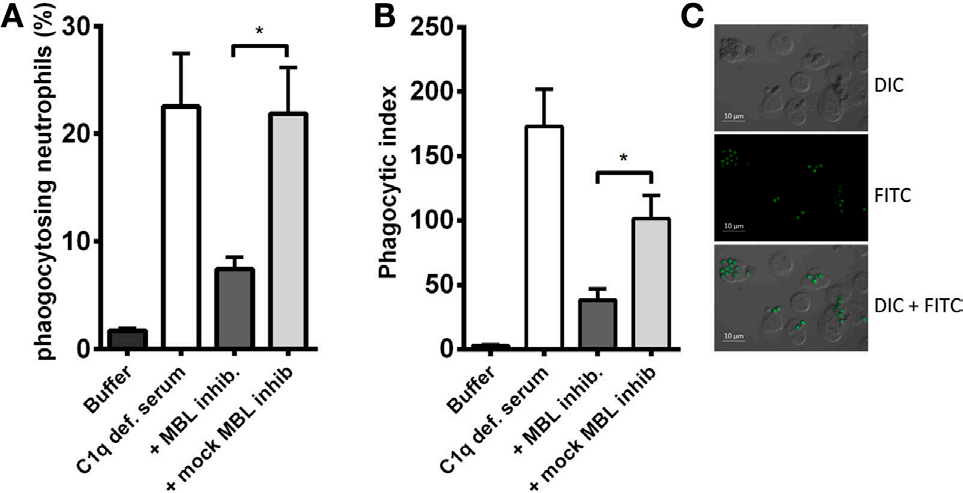
**MBL-mediated opsonophagocytosis of *A. fumigatus* in C1q-deficient serum**. FITC-conjugated *A. fumigatus* conidia (1 × 10^7^ conidia/ml) were opsonized with C1q-deficient serum including MBL inhibitor or MBL mock-inhibitor. Opsonized conidia were mixed with isolated human neutrophils in a ratio of 5:1, and phagocytosis was quantified by flow cytometry. **(A)** The percentage of phagocytizing neutrophils. **(B)** Phagocytic index (percentage of phagocytizing neutrophils × MFI). **(C)** Fluorescence and DIC microscopy image of neutrophils phagocytizing *A. fumigatus-*FITC. Results represent the means of three independent experiments ± SD, *p ≤ 0.05 (two-tailed Student’s *t*-test). MFI = mean fluorescence intensity.

A crucial function of complement is to facilitate phagocytosis. Therefore, we tested whether the MBL-driven complement activation in C1q-deficient serum had an impact on the neutrophilic uptake of *A. fumigatus*. FITC-conjugated conidia were opsonized with C1q-deficient serum mixed with the MBL inhibitor, and afterward phagocytosis by isolated human neutrophils was assessed. We found that the opsonization potential of C1q-deficient serum decreased as a result of MBL inhibition (Figure 6); both the percentage of phagocytizing neutrophils and the phagocytic index (the amount of conidia per neutrophil) were significantly reduced upon MBL inhibition (Figures 6A,B). Figure 6C presents a visual impression of the phagocytic process shown with fluorescence and differential interference contrast (DIC) imaging.

### The Role of MBL in Umbilical Cord Serum

**Figure 7:**
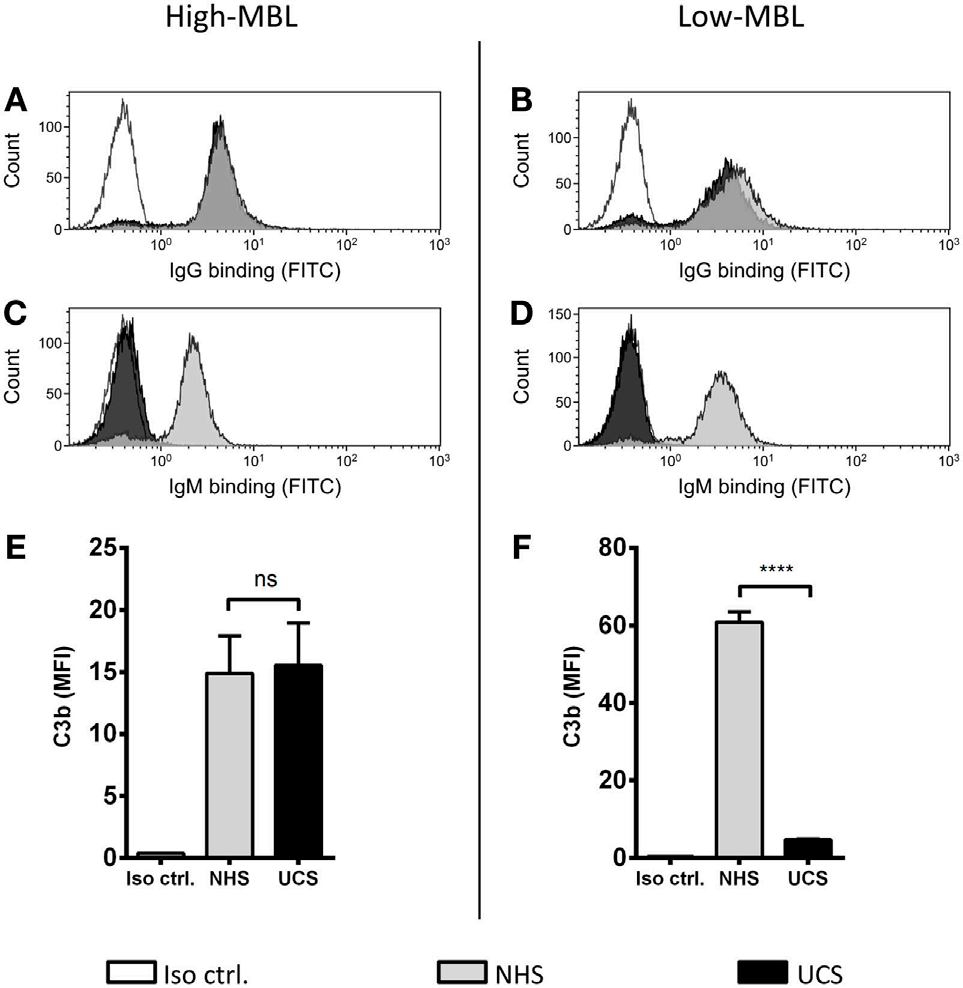
**MBL-mediated complement activation in the absence of IgM binding**. NHS and UCS pools with “high” and “low” contents of MBL were mixed with *A. fumigatus* (1 × 10^7^ conidia/ml) to determine the following components: **(A,B)** IgG binding, **(C,D)** IgM binding, **(E)** C3b generated from “high-MBL” NHS and “high-MBL” UCS, and **(F)** C3b generated from “low-MBL” UCS and “low-MBL” NHS. Deposited C3b and bound IgG/IgM were measured by flow cytometry and expressed as mean fluorescence intensity (MFI). Results represent the means of three independent experiments ± SD, ****p ≤ 0.0001 (unpaired Student’s *t*-test).

**Figure 8:**
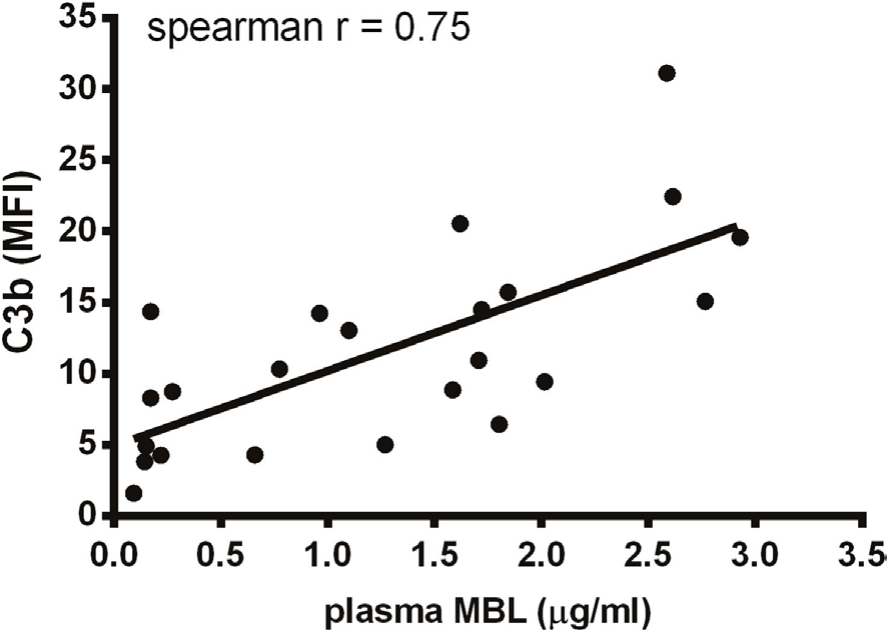
**Correlation between MBL and C3b in umbilical cord serum**. *A. fumigatus* (1 × 10^7^ conidia/ml) was added into UCS samples, and C3b deposition was measured using flow cytometry. MBL levels were measured in the same samples using a single-epitope sandwich ELISA. The two factors – C3b and MBL – were positively correlated (spearman rank = 0.75, *p* < 0.0001, *n* = 23). C3b is expressed as mean fluorescence intensity (MFI) and represent the mean of three independent experiments. MBL measurements were performed in triplicates and are presented in micrograms per milliliter.

We have shown that MBL is an important complement activator in C1q-deficient serum. Our next step was to explore the role of MBL when classical pathway function was compromised due to immunoglobulin insufficiency. Based on MBL serum levels, NHS and UCS samples were divided into serum pools containing either low-MBL levels (~0.4 μg/ml) or high MBL (~2 μg/ml) (Figure S2 in Supplementary Material). The two NHS pools facilitated both IgG and IgM binding, whereas the UCS pools facilitated IgG but not IgM binding (Figures 7A–D). “High-MBL” UCS and NHS showed equivalent levels of deposited C3b, but “low-MBL” UCS mediated significantly less C3b than “low-MBL” NHS (Figures 7E,F). Thus, despite significant IgG binding in UCS, the absence of IgM appeared to affect the classical pathway to an extent that made MBL the central complement activator on *A. fumigatus*. These results were supported by the existence of a strong positive correlation between MBL levels and C3b deposition in UCS (Figure 8).

### C1q and MBL Inhibition in Normal and Umbilical Cord Serum

**Figure 9:**
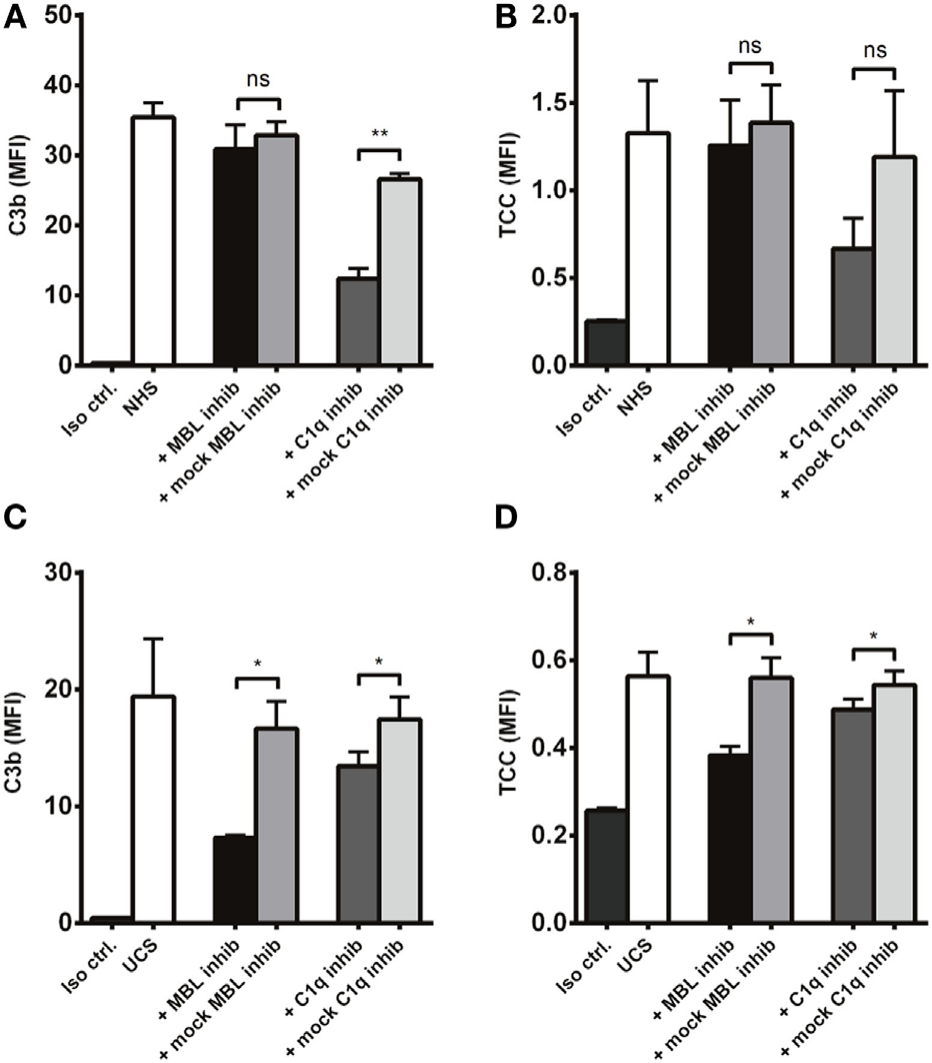
**Inhibition of MBL in umbilical cord serum**. *A. fumigatus* (1 × 10^7^ conidia/ml) was applied into **(A,B)** NHS **(C,D)** UCS to estimate the effect of C1q and MBL inhibition. Complement products were measured by flow cytometry and expressed as mean fluorescence intensity (MFI). Results represent the means of three independent experiments ± SD, *p ≤ 0.05. **p ≤ 0.01 (one-way ANOVA, Bonferroni’s multiple comparison test).

To further differentiate C1q and MBL as complement activators on *A. fumigatus*, we compared the effect of C1q and MBL inhibition in NHS and UCS. C1q inhibition significantly reduced C3b deposition in NHS, while MBL inhibition had no effect (Figure 9A). There was a similar pattern regarding the formation of TCC (Figure 9B). In UCS, however, MBL inhibition reduced complement activation two to three times more than C1q inhibition (Figures 9C,D).

### C1q and MBL Inhibition in Serum from Immunocompromised Patients

The results presented up to this point were generated using model systems with C1q-deficient serum and UCS to explore the possible behavior of complement in patients with a low supply of anti-*A. fumigatus* Abs. As a continuation, we tested whether the proposed role of MBL as the main activator under such conditions was applicable to a more authentic model. For this purpose, we used sera from three patients suffering from different immune disorders. Table 1 shows the diagnosis and immunoglobulin levels as reported in the clinical records. In addition the table shows our measurements of the binding of IgG and IgM to *A. fumigatus*; we assigned the measurements with ratings from – to +++, according to a comparison with the binding levels observed in the previously applied NHS pool. These measurements, combined with the reduction in C3b caused by C1q or MBL inhibition, provided the following information: MBL-initiated activation accounted for approximately 70% of the total complement activation in the samples with low IgG/IgM binding (61 and 75%), whereas the sample with abundant IgG/IgM binding mainly activated complement *via* C1q (67%). Again IgM seemed to play a central role as the patient with no IgM binding (X-linked agammaglobulinemia) had low classical pathway activity despite measurable IgG binding (together C1q and MBL inhibition did not add up to 100% as they were not applied simultaneously and possibly due to the alternative pathway amplification).

## Discussion

Complement is a crucial part of the innate immune system and has been shown to participate in the defense against *A. fumigatus* (12), but the roles of the three complement pathways have never been fully established. Through *in vitro* experiments, we approached this query from two angles – the immunocompetent and the immunocompromised situation – as we found this particularly important for studying an opportunistic pathogen like *A. fumigatus*.

Initially, we established that alternative pathway overall strongly boosted complement activation on resting *A. fumigatus* conidia. The importance of alternative pathway on *A. fumigatus* was already described in studies made 30–40 years ago (13, 14), but as opposed to those observations we did not find alternative pathway capable of initiating the cascade. In our hands, classical and/or lectin pathway were needed to generate the first C3 convertases, which we demonstrated using Mg/EGTA on normal serum, thereby excluding the lectin and classical pathways, and by using C2-deficient serum. This is also in contradiction to the previously reported C2-bypass mechanism of activation on *A. fumigatus* (15). This might be explained by differences between *A. fumigatus* strains, an issue that could have been addressed by incorporating multiple strains; however, this was beyond the scope of the present study.

Previously, MBL (16), ficolin-2 (17), and C1q (18) have been shown to bind *A. fumigatus*, which we confirmed in NHS. As we progressed to characterize the complement initiation process, we found C1q to be the main conductor of activation, while MBL and ficolin-2 did not contribute significantly. Recently, another *in vitro* study reached a similar conclusion showing that C1q-mediated complement activation led to neutrophilic phagocytosis of *A. fumigatus* (19), which underlines classical pathway as the likely route of complement activation under normal immunocompetent conditions.

This situation, however, is different from the medical background of most IPA patients; hence, we wanted to mimic the limitation of Abs that exists as a secondary complication in many immunocompromised patients. Classical pathway would probably be disadvantaged by a low antibody content, which made C1q-deficient serum a relevant matrix to study. We found that MBL (but not ficolin-2) was able to activate complement on behalf of C1q and furthermore able to mediate opsonophagocytosis.

The next step was to simulate the actual inadequacy of Abs, which we pursued in serum from umbilical cords. We found that UCS did not facilitate any IgM binding to *A. fumigatus* and that a pool of UCS with a low-MBL content had substantially decreased complement activation – a relation not seen in NHS with low-MBL content. The clear positive correlation between MBL levels and C3b deposition in UCS supported this observation. It is noteworthy that IgM predicted the outcome of classical pathway activity since IgG binding from UCS did not resurrect the pathway. We made a similar observation in serum from an X-linked agammaglobulinemic patient in IgG replacement therapy (IVIG); despite IgG binding to *A. fumigatus*, the absence of IgM positioned MBL as the main complement activator instead of C1q. Unfortunately, only three immunoglobulin-insufficient patients were eligible for this investigation. Therefore, conclusions based on these experiments alone should be considered with some caution, but collectively the UCS samples and the patient samples give a good indication of the general complement mechanisms in a compromised immune system.

A clinical study showed that IPA patients had lower concentrations of MBL in their serum compared to febrile immunocompromised controls and that there was a higher frequency of MBL deficiency among the IPA patients (20). Other clinical studies have shown that patients with chronic forms of pulmonary aspergillosis have lower levels of high-order oligomeric MBL (21, 22). Our findings could very well explain the outcome of these different clinical studies and substantiate MBL as a possible future drug-component for treatment and prophylaxis against *A. fumigatus*.

As a proof of concept, it has been shown that corticosteroid-immunosuppressed mice infected with *A. fumigatus* had an enhanced chance of survival after MBL-treatment (23). In this context, it is important to mention another study showing that non-immunosuppressed MBL-knockout mice were less susceptible to systemic *A. fumigatus* infection compared to wild-type mice (24). It was suggested (not shown) by the authors that the wild types did not die due to *A. fumigatus*, but from neutrophilic infiltration causing detrimental tissue-damage. Nevertheless, it shows that MBL is a double-edged sword, and care must be taken in potential future MBL-treatment. For the time being, MBL screenings could possibly be a useful tool in the clinic to evaluate the risk of *A. fumigatus* infection in immunocompromised patients, especially considering that around 10% of the Caucasian population carries gene variants that cause different MBL-deficient states (25).

Finally, our findings also suggest IgM-replacement therapy as another treatment option, since classical pathway seemed to be highly dependent on IgM to initiate complement on *A. fumigatus*. IgM-enriched intravenous IgG (IVIG) has been shown to possibly benefit patients with Gram-negative septic shock (26) and perhaps this applies to IPA patients as well.

## Conclusion

In a competent immune system, *A. fumigatus* conidia are eliminated before an invasion of the pulmonary tissue can occur. However, in the absence of a well-functioning defense, *A. fumigatus* can cause severe infections and ultimately death. C1q appears to activate complement on *A. fumigatus* in the immunocompetent state, but many immunocompromised patients have low antibody levels and according to our findings this seems to position MBL as the key activator of complement instead of C1q. Possibly this explains why clinical studies have shown that low-MBL levels are a risk factor of *A. fumigatus* infections. With this study, we contribute to a deeper insight into the mechanisms of how complement combats *A. fumigatus* infections under healthy and especially immunocompromised circumstances.

## Author Contributions

AR: study design, experimental work, data interpretation, drafting the article, and final approval. NG and KP: study design, data interpretation, critical revision of the article, and final approval. M-OS: data interpretation, critical revision of the article, and final approval. GS: critical revision of the article and final approval. PG: study design, critical revision of the article, and final approval.

## Conflict of Interest Statement

The authors declare that the research was conducted in the absence of any commercial or financial relationships that could be construed as a potential conflict of interest.

## Abbreviations

IPA, invasive pulmonary aspergillosis; mAb, monoclonal antibody; MBL, mannose-binding lectin; MFI, mean fluorescence intensity; NHS, normal human serum; pAb, polyclonal antibody; PRM, pattern-recognition molecule; UCS, umbilical cord serum.
