# Supplementary figures and images for: Complementary Roles of the Classical and Lectin Complement Pathways in the Defense against Aspergillus fumigatus
Source: Front Immunol. 2016 Nov 3;7:473. doi: 10.3389/fimmu.2016.00473 (PMC5093123; doi:10.3389/fimmu.2016.00473)

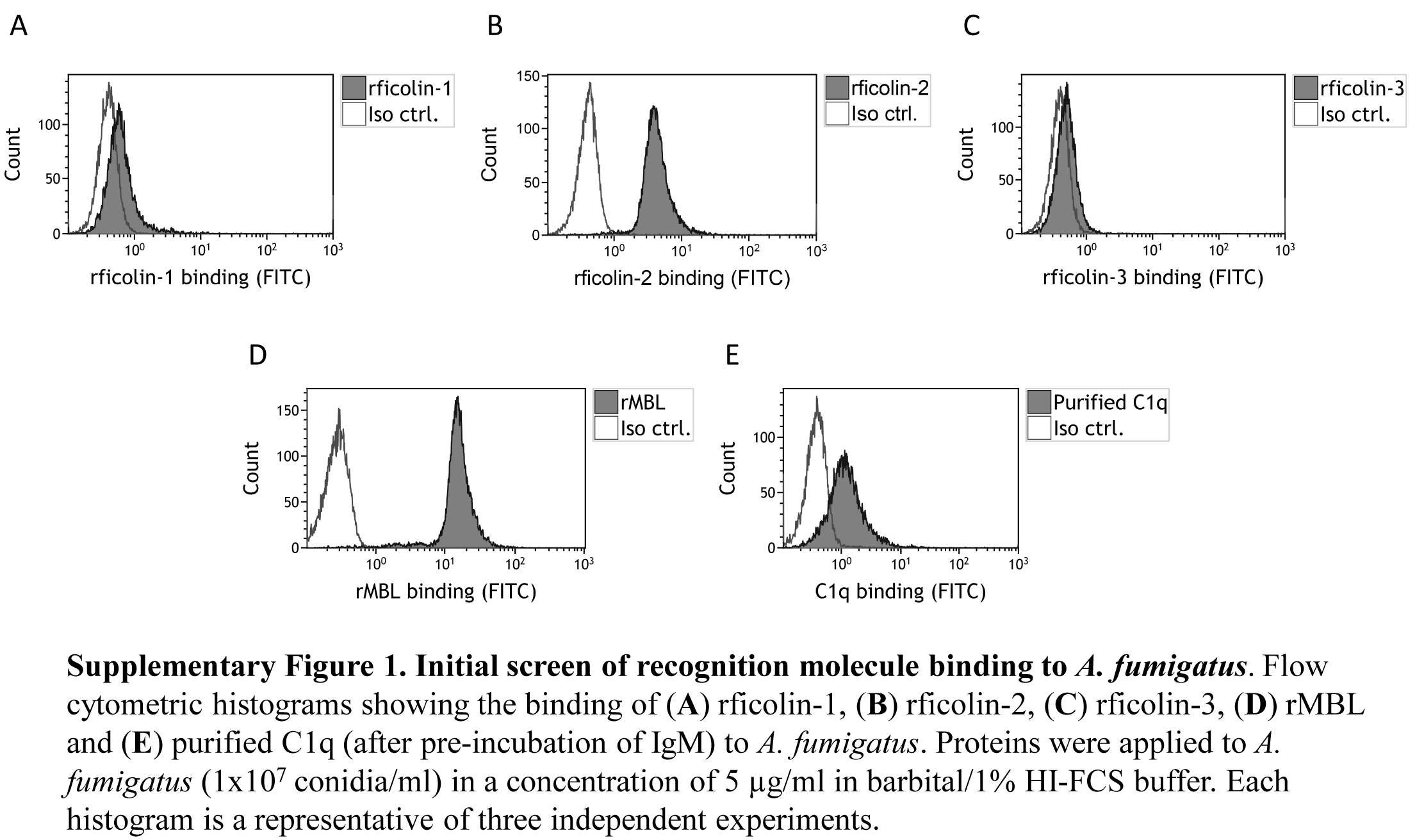

Supplement: Supplementary file 1 [file Image_1.TIF]

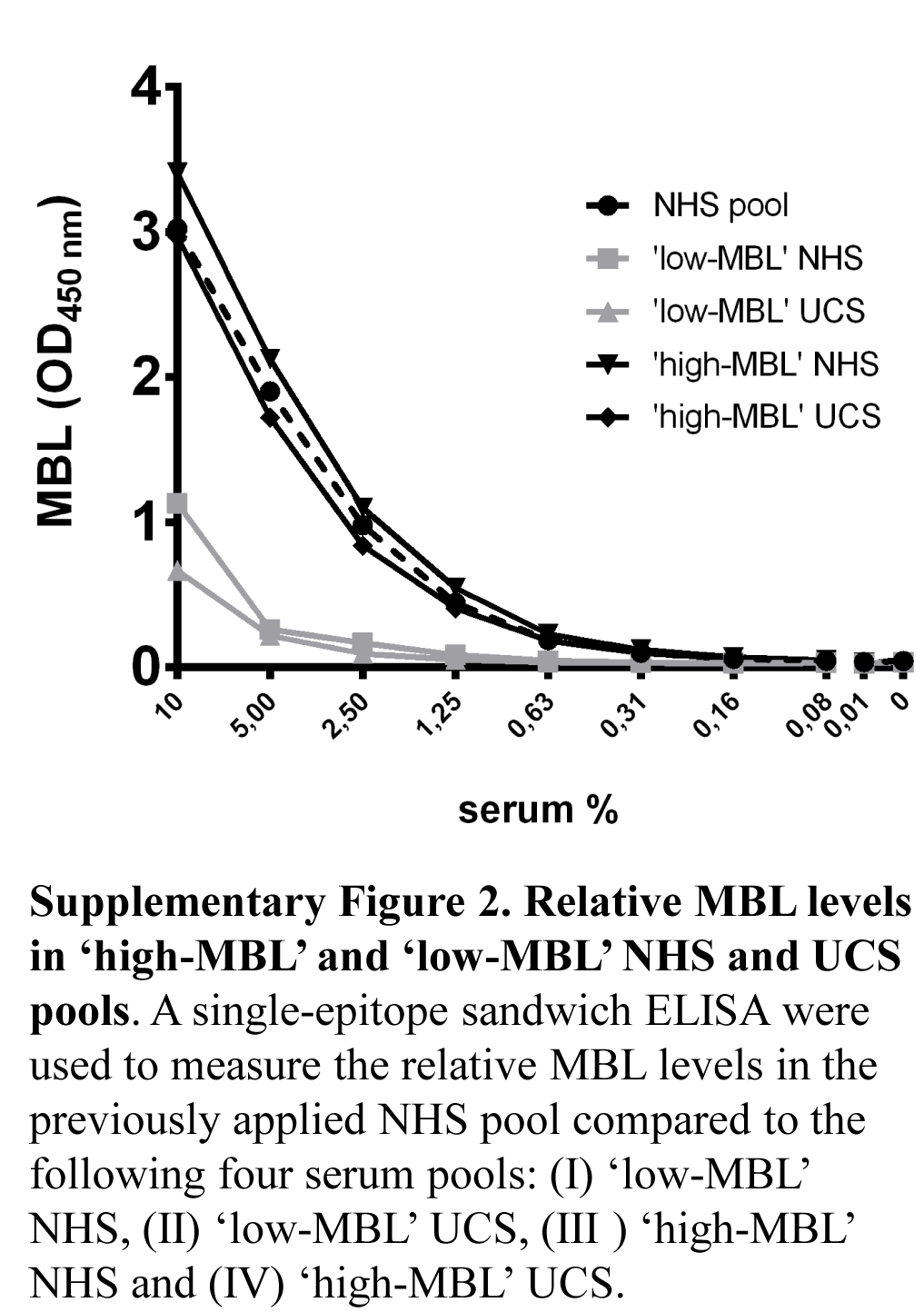

Supplement: Supplementary file 2 [file Image_2.TIF]
